# Enhanced, coordinated conservation efforts required to avoid extinction of critically endangered Eastern Pacific leatherback turtles

**DOI:** 10.1038/s41598-020-60581-7

**Published:** 2020-03-16

**Authors:** The Laúd OPO Network, Marino Eugenio Ábrego, Nicolás Acuña-Perales, Joanna Alfaro-Shigueto, Jorge Azócar, Ana Rebeca Barragán Rocha, Andrés Baquero, Alejandro Cotto, Jodie Darquea, Nelly de Paz, Miguel Donoso, Peter H. Dutton, Luis Fonseca, Velkiss Gadea, Débora García, Meritxell Genovart, Astrid Jimenez, María del Rosario Juárez, Karla Cecilia López Sánchez, Jeffrey C. Mangel, Mayra Leticia Martínez Suzano, Cristina Miranda, Enrique Ocampo, Ana Ordaz Becerra, Clara Ortiz-Alvarez, Frank V. Paladino, Andrea Pasara-Polack, Sergio Pingo, Rotney Piedra Chacón, Javier Quiñones, Juan M. Rguez-Baron, Jorge Carlos Salas Jiménez, Heydi Salazar, Pilar Santidrián Tomillo, Adriana Laura Sarti Martínez, James R. Spotila, Alejandro Tavera, Jose Urteaga, Felipe Vallejo, Elizabeth Velez, Bryan P. Wallace, Amanda S. Williard, Patricia M. Zárate

**Affiliations:** 1 Ministerio de Ambiente de Panamá, Universidad Marítima Internacional de Panamá, Panama City, Panamá; 2 ProDelphinus, Lima, Perú; 3 Facultad de Biologia Marina, Universidad Científica del Sur, Lima, Perú; 4 Instituto de Fomento Pesquero, Valparaíso, Chile; 5 Kutzari, Asociación para el Estudio y Conservación de las Tortugas Marinas, México City, México; 6 Equilibrio Azul, Quito, Ecuador; 7 Fauna and Flora International, Managua, Nicaragua; 8 Mundo Ecologico, Quito, Ecuador; 9 Áreas Costeras y Recursos Marinos (ACOREMA), Lima, Perú; 10 World Wildlife Fund-Perú, Lima, Perú; 11 Pacífico Laúd, Quilpué, Chile; 12 NOAA Fisheries, Southwest Fisheries Science Center, La Jolla, CA USA; 13 The Leatherback Trust, Playa Grande, Costa Rica; 14 CEAB (CSIC) and IMEDEA (CSIC-UIB), Blanes, Catalonia, Spain; 15 Centro Tortuguero Playa Cahuitán de la Dirección Regional Frontera Sur, Comisión Nacional de Áreas Naturales Protegidas, Cahuitán, México; 16 University of Exeter, Penryn, Cornwall, UK; 17 Center for Marine Conservation, Purdue University, Fort Wayne, IN USA; 18 Área de Conservación Tempisque, Ministerio del Ambiente y Energía, San José, Costa Rica; 19 Oficina de Investigaciones en Depredadores Superiores,Instituto del Mar del Perú (IMARPE), Callao, Perú; 20 JUSTSEA Foundation, Bogotá, Colombia; 21 Department of Biology and Marine Biology, University of North Carolina Wilmington, Wilmington, NC USA; 22 Programa Nacional para la Conservación de las Tortugas Marinas, Comisión Nacional de Áreas Naturales Protegidas, México City, México; 23 Drexel University, Philadelphia, PA USA; 24 EIPER, Stanford University, Stanford, CA USA; 25 Kuemar, San José, Costa Rica; 26 Ecolibrium, Inc, Boulder, CO USA; 27 Nicholas School of the Environment, Duke University, Durham, NC USA

**Keywords:** Conservation biology, Population dynamics, Marine biology

## Abstract

Failure to improve the conservation status of endangered species is often related to inadequate allocation of conservation resources to highest priority issues. Eastern Pacific (EP) leatherbacks are perhaps the most endangered sea turtle population in the world, and continue on a path to regional extinction. To provide coherent, regional conservation targets, we developed a population viability analysis and examined hypothetical scenarios describing effects of conservation activities that either reduced mortality or increased production of hatchlings (or both). Under status quo conditions, EP leatherbacks will be extirpated in <60 yr. To ensure a positive, long-term population trajectory, conservation efforts must increase adult survivorship (i.e., reduce adult mortality) by ≥20%, largely through reduction of fisheries bycatch mortality. Positive trajectories can be accelerated by increased production of hatchlings through enhanced nest protection and treatment. We estimate that these efforts must save approximately 200–260 adult and subadult leatherbacks and produce approximately 7,000–8,000 more hatchlings annually. Critically, reductions in late-stage mortality must begin within 5 years and reach 20% overall within the next 10–15 years to ensure population stabilization and eventual increase. These outcomes require expanded, sustained, coordinated, high-priority efforts among several entities working at multiple scales. Fortunately, such efforts are underway.

## Introduction

Species declines driven by human causes have been well-documented across taxonomic groups in recent decades^1^. In some cases, persistent, effective conservation efforts – often coupled with enforced legal protections – have stabilized and even begun to recover populations^2^. Among long-lived marine species such as marine mammals, turtles, birds, and elasmobranchs, there is wide variation in long-term population trends and whether conservation efforts have successfully achieved recovery goals^3–5^. For populations to recover, threats must be reduced to levels that first stabilize, then increase population abundance; however, failure to recover depleted populations often is related to insufficient (i.e., too little for too short a time period) or inefficient allocation of limited resources to conservation actions of highest potential benefit to the target population^6^.

Marine turtles exemplify these issues, given their global distributions and varied conservation status among regional population segments (i.e., regional management units, RMUs; Ref. ^7^). Many RMUs within and among the seven extant marine turtle species are depleted relative to historical abundance because of human-caused mortality, namely direct harvest of turtles and their eggs and incidental capture in fisheries^8^. However, some marine turtle populations are showing signs of recovery following conservation efforts targeting anthropogenic sources of mortality such as harvest of turtles and eggs for human consumption^5^. Marine turtles are long-lived species that often take decades to reach maturity and have several, long-duration life stages that occupy large and varied marine ecosystems^7^. Thus, any efforts to recover depleted populations and safeguard healthy populations against future declines must be focused on reducing the threats with the highest population-level effects and sustained over long periods of time (i.e., decades)^8,9^. In particular, conservation efforts that increase survival of late-stage individuals (i.e., adults and subadults) will provide the greatest population benefit on a per-individual basis^9^.

**Figure 1:**
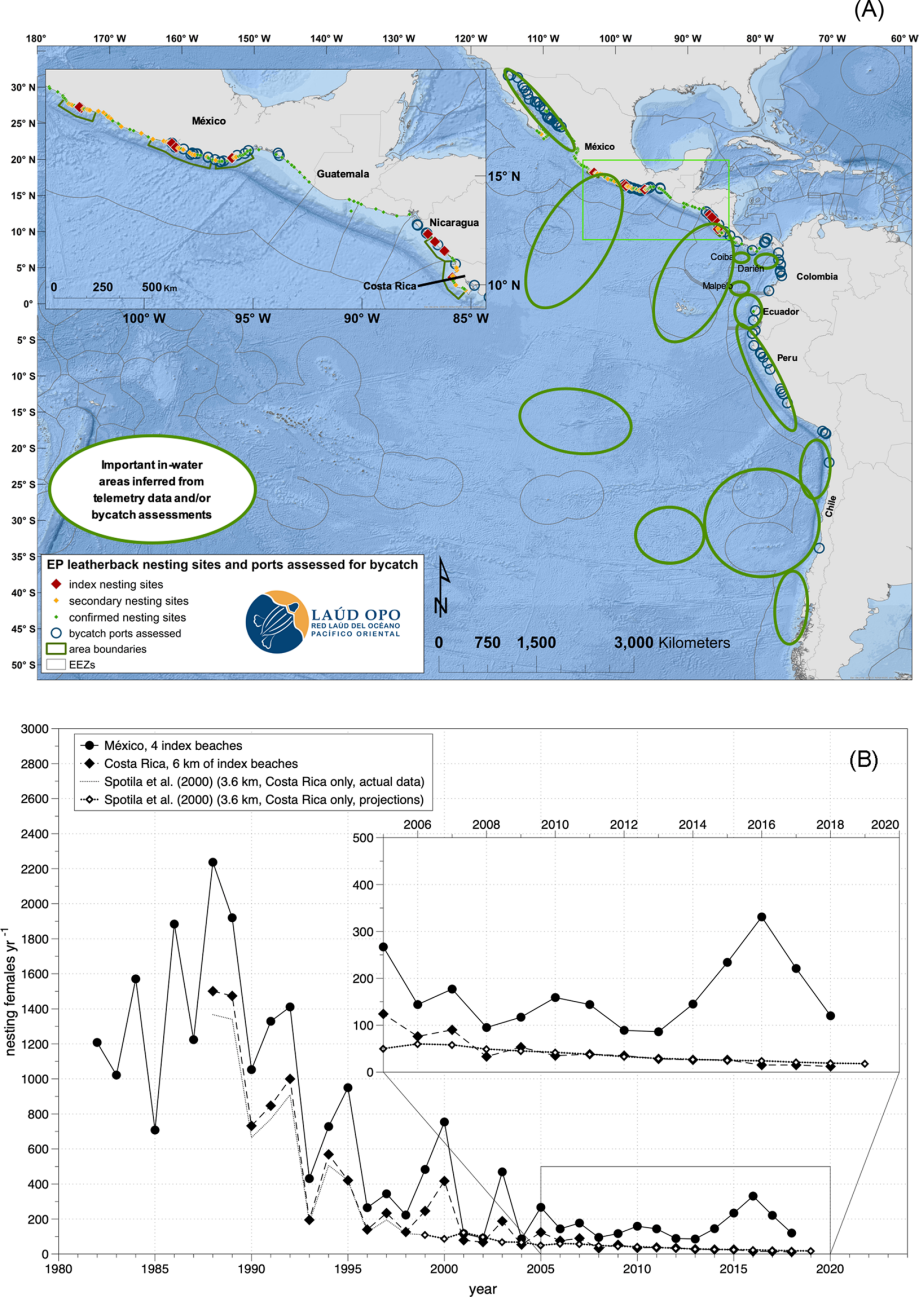
Nesting sites, ports assessed for bycatch, generalized areas of importance (i.e., based on known nesting sites and documented patterns of habitat use and reports of bycatch; refs. ^13–16,23,24,27–31,49^) (**A**) and population trends (**B**) for Eastern Pacific leatherbacks. Index beaches in México, including Mexiquillo (until 2013), Tierra Colorada, Barra de la Cruz, Cahuitán, and Chacahua (since 2011). Nicaragua data not shown because 1) the time series of annual counts did not begin until 2002, after the overall population had already declined ~90%, and 2) abundance values are much lower than in Costa Rica. Map generated in ArcMap 10.2.1 (Environmental Systems Research Institute, Redlands, CA, USA).

One of the most well-known examples of a marine turtle RMU that has declined precipitously in recent decades is the Eastern Pacific (EP) leatherback turtle (*Dermochelys coriacea*)^10^. Leatherbacks have existed around the world for more than 20 million years, and are the sole extant member of the Family Dermochelyidae^11^. Annual counts of nesting turtles and nests on beaches in México and Central America have declined by more than 90% since the 1980s, which qualified EP leatherbacks as Critically Endangered on the IUCN *Red List of Threatened Species*^12,13^ (Fig. 1). Incidental capture in fisheries (i.e., fisheries bycatch) and high levels of egg consumption by humans have been identified as the main drivers of the population decline^10,13–17^, though climate change may have exacerbated the collapse and could pose future obstacles to recovery^18,19^.

In addition to its critical conservation status being well-documented, EP leatherbacks are among the most well-studied marine turtle populations in the world, particularly with respect to aspects of their nesting ecology (see ref. ^20^ for review). This population has been monitored and protected at high-density nesting beaches in México and Costa Rica since the 1980s^14,21^, as well as at other nesting beaches from northern México through Central America in more recent years^22^. Movements, habitat use, and long-distance migrations of adult females^23,24^, as well as hatchling dispersal patterns^25^ have been thoroughly described. In addition, for ~20 years, a large amount of work has been directed to document and reduce interactions between fisheries and leatherback turtles in the East Pacific Ocean^26^. These efforts have been focused mainly in Perú and Chile where high levels of EP leatherback bycatch have been consistently reported^27–31^.

A pair of papers^10,32^ first sounded the alarm about the EP leatherbacks’ decline, with the more recent study presenting a population model (based on data from the Costa Rica nesting population) that projected continued exponential declines and potential extirpation by 2019. Subsequently, the leatherback was first classified in 2000 as Critically Endangered globally on the IUCN *Red List of Threatened Species*^12^, a status that was confirmed for the EP subpopulation by a more recent (2013) Red List assessment^13^. In the nearly two decades since the first models were published, observations from beach monitoring have largely mirrored the predictions of previous models^10,32^, particularly for Costa Rica^19^. Nesting abundance on Mexican beaches has also declined exponentially, though nesting female abundance in recent years has remained substantially higher in México than in Costa Rica (Fig. 1). However, the vast majority of published research to date has focused on the Costa Rica nesting population, and all existing EP leatherback population models have focused solely on the Costa Rica dataset^10,15,18,19^; thus, there has been no previous attempt to combine and analyze regionally available population information as a single population. Genetic studies^33^ and satellite telemetry^23,24^ have shown that EP leatherback turtles belong to a single metapopulation that is subject to similar environmental and anthropogenic drivers^34^. For these reasons, a combined analysis is warranted and would provide holistic results applicable to the entire population.

Decades of research and conservation work have yet to yield signs of population stabilization, let alone recovery of EP leatherbacks^35^. In response, a regional network of researchers, managers, and conservationists—*la Red para la Conservación de la Tortuga Laúd en el Océano Pacífico* (Red Laúd OPO, or Eastern Pacific Leatherback Conservation Network, hereafter Laúd OPO Network)—created a regionally coherent action plan for preventing extinction and recovering the EP leatherback population^35^. Here, we present the results of the first comprehensive population viability analysis for the EP leatherback population (>90% of overall nesting female abundance), using information inferred from capture-recapture analyses, and nesting and bycatch data. In addition to providing an update on the EP leatherback population status nearly 20 years after the original population model^10^, we constructed our model to estimate possible population responses to future scenarios of conservation investments. Thus, our ultimate goal was to provide coherent, population-level targets for regional conservation activities on nesting beaches and in marine habitats throughout the region so that collective efforts will ultimately promote population recovery.

## Methods

We first derived demographic parameters for the different subpopulations in the area based on empirical data, where available, or estimates, where necessary.

### Reproductive parameters

To estimate reproductive parameters, we used data from the nesting populations in México and Costa Rica for seasons between 2004–2005 and 2016–2017 and all available years for Nicaragua (2004–2005 to 2015–2016 for nesting activities and 2011–2012 to 2015–2016 for hatching success). To estimate fecundity, we calculated annual means (±SD) of (1) number of eggs in a clutch, (2) estimated clutch frequency (ECF) (i.e., the number of clutches produced per female per season), (3) emergence success (i.e., proportion of eggs that produce hatchlings that emerge from nests and reach the sand surface), (4) proportion of hatchlings per female that reached the water, and (5) primary sex ratios. We considered that the proportion of hatchlings reaching the water in Nicaragua would equal that of Costa Rica and that primary sex ratios in México and Nicaragua mirrored those of Costa Rica. Sex ratios had been previously estimated for Costa Rica^19^ using the temperature-dependent sex determination curve described previously^36^ for this population. Since beach protocols vary among countries, we used different equations to estimate annual production of hatchlings per female.

For the Costa Rican/Nicaragua subpopulation we used the following equation:

1 $${\rm{H}}={\rm{f}}\ast {\rm{es}}\ast {\rm{w}}\ast {\rm{fem}},$$

where *H* is the number of female hatchlings produced per nesting female that reach the water in a season, which is a product of: *f*, the average number of eggs produced in a season per female (obtained multiplying the average number of eggs by ECF); *es* mean emergence success; *w* mean proportion of hatchlings that reach the water after emergence^37^, and; *fem*, the proportion of hatchlings that are female hatchlings.

For México, we used:

2 $${\rm{H}}={\rm{f}}\ast {\rm{hw}}\ast {\rm{fem}},$$

where *H, f* and *fem* are as above and *hw* is the number of hatchlings that reach the water in relation to the number of eggs in a clutch. Because all clutches are relocated in México, the exact number of hatchlings that make it to the water in relation to the number of eggs laid is known.

### Annual survival, remigration, transient, and capture probabilities

As adult female individuals are first marked only when they visit beaches to breed, we could not estimate first year, juvenile, and subadult survival based on capture-recapture data. We considered first year survival S_1_ = 0.063, based on Ref. ^32^. For juvenile survival we conservatively estimated the juvenile survival probability that would maintain population stability (S_2_ = 0.500), assuming adult and subadult survival probabilities without anthropogenic mortality (>0.90; i.e., corresponding to stable or increasing leatherback populations; ref. ^38–40^) and under the current rates of fecundity, transient, and recruitment probabilities. We considered that survival of subadults was the same as that of adults because of the large body size attained by subadults^41^, and relatively small adult body sizes documented for the EP population^42^. Adult survival estimates, probabilities of breeding for different remigration intervals, and transient probabilities (i.e., individual that is only seen during one nesting season and never thereafter) were estimated for this study (Table 1).

**Table 1:** Mean estimates used in the projection of current conditions obtained from this and previous studies.

Parameter	Costa Rica	México	Source
First year survival, S1	0.063	0.063	ref. ^32^
Juvenile survival, S2	0.500	0.500	This study
Subadult and adult survival, S3	0.788	0.705	ref. ^57^; This study
Breeding probability the year after breeding, B1	0.005	0.003	This study
Breeding probability 2 years after breeding, B2	0.272	0.169	This study
Breeding probability 3 years after breeding, B3	0.540	0.379	This study
Breeding probability ≥4 years after breeding, B4	0.681	0.550	This study
Probability of being transient	0.131	0.279	This study
Fecundity (eggs/female/year)	403	390	This study
Current egg harvest level	1%	4.2%	This study
Survival to water (from egg)	0.31	0.47	ref. ^37^; This study
Sex ratio	0.840	0.840	ref. ^19^, based on the TSD-curved developed by ref. ^36^

**Figure 2:**
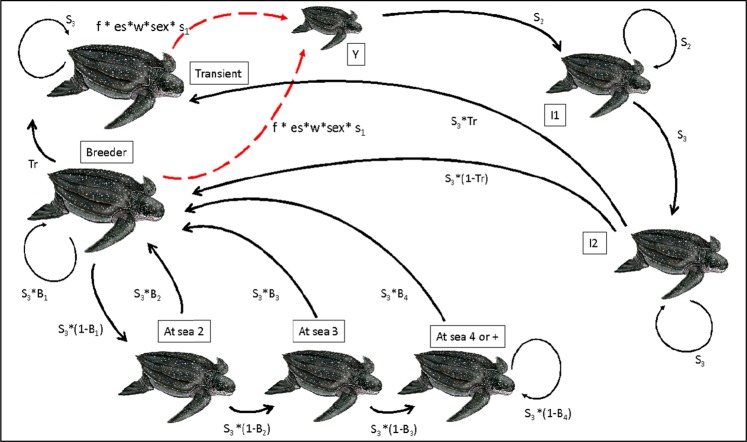
Life cycle of the eastern Pacific leatherback turtle based on eight life stages. Where “Y” stands for yearlings, “I1” juveniles until age of three, “I2” subadults between three and twelve years old, “Breeder” nesting females, “Transients” females marked in one nesting season and never seen again, “At sea 2” turtles at sea two years after nesting, “At sea 3” three years after nesting, “At sea 4+” four or more years after nesting. “F” eggs per female per season, “es” emerging success, “w”, survival to the water, “sex”, proportion of females, “Tr” probability of being a transient, “B_i_” probability of breeding at year i, “S_1_” survival in the first year of life, “S_2_” survival between years 1–3, “S_3_” survival of adults. The equation for hatchling production (f *es*w*fem) corresponds to the Costa Rican subpopulation. Hatchling production for México was: H = f*hw*fem, where “hw” was the number of hatchlings that reached the water in relation to clutch size.

Stages referred to below appear in Fig. 2. First year and juvenile survival and fecundity, survival to the water and sex ratio were based on published estimates, while estimates for transient probability, survival probability and breeding propensity probabilities come from models with constant values to derive the variance-covariance matrix (Supplemental Table 1: models 23 and 5, for México and Costa Rica, respectively).

We estimated annual survival rates of adult female turtles and remigration probabilities based on capture-mark-recapture (CMR) data for the two long-term monitoring projects where turtles have been consistently tagged over time. These data include all index beaches in the EP: the four primary index nesting beaches in México (Mexiquillo, Tierra Colorada, Cahuitán y Barra de la Cruz) and the one site in Costa Rica (three beaches of Parque Nacional Marino Las Baulas: Playa Grande, Playa Ventanas, and Playa Langosta) (Fig. 1). The monitoring project at México started in 1980 and marked turtles initially with metal and plastic tags, and then with Passive Integrated Transponder (PIT) tags beginning in 1996–1997. Monitoring at Costa Rica started in 1993–1994 and turtles have always been marked with PIT tags.

For both subpopulations, we created a database with individual encounter histories for nesting females. We first assessed the fit of our data with a Cormack-Jolly-Seber model using U-care^43^. Based on the goodness of fit test (GOF) and the main sources of overdispersion, we built multi-event models separately for each database. Then, using program E-Surge^44^, we estimated annual survival and remigration probabilities, probability of being a transient animal, and detection probabilities for both subpopulations. Within the multi-event, capture-recapture framework, models hold two levels in the capture-recapture data: (1) the field observations, called “events,” encoded in the capture histories, and (2) the “states” defined to match the biological questions that can only be inferred^45^. For all analysis we defined two events: “0” for those occasions when an individual had not been seen and “1” for those occasions when an individual had been seen alive and nesting. Capture-recapture models included twelve biological states in relation to the breeding state (Fig. 2), the presence of transients and a hypothetical maximum interbreeding interval of 10 years: breeder alive (B); alive at sea (1–10 states depending on the time spent at sea without breeding) and dead (D), this last state being generally non-observable. The initial state in our models was always B. Transitions between states were modelled in a three-step approach: transient probability, survival, and recruitment probability (Supplementary Table 1).

Because two different types of tags were used in México (first flipper tags, then PIT tags), we first conducted an analysis for the entire study period to estimate the loss rate for each tag type^46^. We considered three different groups depending on the type of tag and tested the hypothesis that individuals with different tags had different survival probabilities. Plastic tags were more easily lost (~60% lost annually) than metal tags, but metal tags also experienced a significant loss rate (~30% lost annually). Therefore, considering these results together with the fact that PIT tags have a retention rate of nearly 100%^47^, we restricted CMR analysis to years in which leatherbacks were PIT tagged. Thus, our dataset included data from 1996–97 to 2016 in México and from 1993–94 to 2015 in Costa Rica.

For both subpopulations, we tested whether demographic parameters were constant (.) or changed over time (t), and we also tested the effect of the Multivariate ENSO Index (MEI) (https://www.esrl.noaa.gov/psd/enso/mei/). Model selection relied onthe Akaike Information Criterion corrected for overdispersion and for small sample sizes (QAICc)^48^.

### Bycatch estimates

In order to contextualize results of the population model, we compiled existing bycatch data into a single, regional bycatch database. This data compilation allowed us to generate region-wide estimates of average annual number of leatherbacks caught and killed in fishing gear in the EP region. We first used recently published data from rapid bycatch assessments (i.e., port-based interviews) focused on net gear in Ecuador, Perú, and Chile^30^ to estimate leatherback bycatch interactions and mortality by port, by country, and across all three countries. We then supplemented these data with published and unpublished bycatch information from Laúd OPO Network partners collected previously as well as new data collected during the past two years, particularly in Perú and Chile. In these latter cases, bycatch data were collected via on-board observers (e.g., Instituto del Fomento Pesquero in Chile^31^) as well as rapid bycatch assessments (e.g., ProDelphinus^29,30^, ACOREMA in Perú^49^). For these data, we used mortality rates^17^ of leatherbacks (i.e., the percentage of bycatch interactions that result in mortality) as 5% for longlines and 33% for nets if mortality information was unavailable.

We note that these bycatch data predominantly come from small-scale, national-level fisheries, specifically net gear. Very little information is available on leatherback bycatch interactions with industrial-scale fishing gear (e.g., longlines targeting tuna and other pelagic species) in high-seas areas in the Pacific Ocean under multi-national jurisdiction of regional fisheries management organizations^17^, specifically the Inter-American Tropical Tuna Commission (IATTC), whose convention area overlaps with the EP leatherback distribution^8^.

### Population model

We formulated a matrix population model to assess the population growth rate of EP leatherback turtles under current and possible future conditions. The model followed a pre-breeding census format and was based only on females. As demographic parameters were different between México and Costa Rica (see results), we used a 16-stage stochastic population model, with eight stages for each of these subpopulations. The eight stages considered for each population (similar to those used in previous EP leatherback models; refs. ^18,19^) are shown in Fig. 2 and were:

- stage 1 from the time hatchlings reach the water until age 1,
- stage 2 for juveniles between year 1 and year 3,
- stage 3 for subadults between ages 3 and 12,
- stage 4 for breeding turtles,
- stage 5 for turtles at sea 2 year after breeding,
- stage 6 for turtles at sea 3 year after breeding,
- stage 7 for turtles at sea 4 or more years after breeding,
- stage 8 for turtles that breed only once.

As we had no data to estimate demographic parameters for Nicaragua (2.4% of total population) (Fig. 1), we used a conservative approach and considered that individuals from this subpopulation also shared the same demographic parameters with those from Costa Rica. All model projections were developed and executed in R (http://cran.r-project.org).

We first projected the deterministic population growth rate λ (largest eigenvalue of the population matrix^50^) over 100 years using a deterministic model that included the mean values of the estimated vital rates. Many vital rates used in the model were derived from the present study, but some were obtained from previous work (Table 1). To initialize the models, we used the mean estimate of current population size from the most recent five years of data (2011–2015; average = 176 females per year), and the mean number of hatchlings reaching the water in both subpopulations.

We then constructed a stochastic model that accounted for parameter uncertainty and annual variability in vital rates, to estimate the stochastic population growth rate and the probability of extinction under current and possible future conditions. To do so, we selected random values for survival and fertility rates from beta distributions at each projection and in each year of simulations, using the estimated mean, and sampling and process variance values^51^. We did not consider density-dependence in our model. Models were run using Monte Carlo simulations for ‘100 years’ and ‘1000 population’ trajectories.

### Conservation scenarios

We projected EP leatherback population trajectories under present-day scenarios considering the current impact of fisheries bycatch, egg harvest, and the positive effects of current conservation activities on nesting beaches and in marine habitats, and then under hypothetical scenarios of increasing survival rates of different stages that would result from increased conservation interventions on nesting beaches and in fisheries. The goal of this exercise was to determine the current and future conservation status of the population, as well as the types and degrees of additional conservation efforts needed to stabilize and (eventually) recover the population. This approach can provide regionally coherent, tangible targets to guide collective and individual conservation efforts throughout the Eastern Pacific region.

We evaluated effects of conservation activities that either reduced mortality or increased production of hatchlings (or both) on population growth rate. For mortality reduction, we tested different scenarios of increased adult and subadult survivorship rates, which we assumed to be a proxy for reduction in bycatch mortality. This assumption is justified because harvest of nesting females has essentially been eliminated^14^, and there appears to be no other significant sources of late-stage mortality currently affecting this population other than bycatch^8,13^. In all scenarios, annual survival of non-breeders was considered to be equal to the survival of breeders, because we assumed that environmental stochasticity equally affected the two groups. We ran scenarios at status quo survivorship rates, and with increases of 5%, 10%, and 20%, corresponding to equivalent hypothetical reductions in bycatch mortality.

For increased production of hatchlings, we evaluated effects of complete elimination of egg harvest (current levels are 4.2% and 1% for México and Costa Rica, respectively) and increased hatching success resulting from methods to produce more favorable conditions for developing embryos (e.g., lower temperatures, higher moisture content; ref. ^52^). In addition, we analyzed the effect of relocation of egg clutches at risk of erosion and tidal inundation under natural conditions or human effects in high-traffic areas to assess the effectiveness of current conservation practices conducted on the beach. However, because efforts on nesting beaches have been ongoing for decades on all major nesting beaches, the potential for increased hatchling production due to relocation is fairly limited.

We also evaluated the potential effects of head-starting young leatherbacks; i.e., raising hatchlings in captivity for one year before releasing them with the presumed benefit of increased first-year survivorship and thus hypothetically improved long-term survival^53^. For this scenario, which was entirely speculative, we estimated that 50 individual hatchlings could be head-started in the Costa Rica population, which meant applying year-one survivorship rates to 50 individuals per year. We note that while husbandry techniques for leatherbacks have improved greatly to support laboratory-based research projects^41^, there currently are tremendous costs and logistical challenges as well as relatively limited success related to raising leatherbacks in captivity in sufficient numbers to use head-starting as a viable conservation tool. Nonetheless, considering the dire status of the population presently, and the desire to explore the full range of possible options, we included scenarios with head-starting among our model runs for to estimate potential benefits to the population.

We performed model runs under a variety of combinations of the above factors—i.e., changes in late-stage survivorship, changes in beach protection and hatchling production, and head-starting—to identify types, combinations, and extents of conservation interventions that promoted stable and increasing population trends. Under all scenarios, we estimated the mean stochastic population growth rate (λ_*s*_) over a short and relevant time horizon of 100 years from 1000 projections, together with 95% confidence intervals.

## Results and Discussion

### Reproductive parameters

Three-quarters (75.4%) of total region-wide nesting activities between 2004 and 2015 occurred in México, while the remainder occurred in Costa Rica (22.2%), and Nicaragua (2.4%). In México and Costa Rica 70% and 68% of nesting activities, respectively, occurred on index nesting beaches (i.e., those regularly monitored within and among nesting seasons). The remainder of nesting (~30%) occurred on secondary beaches (i.e., those with lower abundance and monitored less frequently than index beaches). In the last five years of the available nesting data (2011–2015), ~1,050 clutches per year were laid in México, Nicaragua, and Costa Rica, which corresponds to 176 nesting females per year. Reproductive investment was similar between subpopulations. The average number of eggs per clutch was 66.0 ± 16.5 in México and 66.0 ± 16.6 in Costa Rica. The estimated clutch frequency was 5.9 ± 2.2 and 6.1 ± 3.1 in México and Costa Rica, respectively. Therefore, the estimated number of eggs produced in a season per female was 403 eggs in Costa Rica and 390 eggs in México (Table 1). The estimated number of hatchlings produced per female that reached the water in a season was 181 hatchlings in México and 123 hatchlings in Costa Rica. We assumed that the number of hatchlings per female that reached the water in Nicaragua was the same as in Costa Rica.

### Annual survival, remigration, transient and capture probabilities

GOF tests were statistically significant (^c = 3.09 and 6.12, for México and Costa Rica, respectively) due to the presence of transients (i.e., individuals having a zero probability of survival after their initial capture^54^) and ‘trap heterogeneity’ (i.e., individuals in a population are not equally ‘catchable’^55^). Thus, all our models for both subpopulations included age and trap awareness, and we corrected for the remaining overdispersion with a ^c = 1.638 and ^c = 3.053 for México and Costa Rica, respectively.

**Figure 3:**
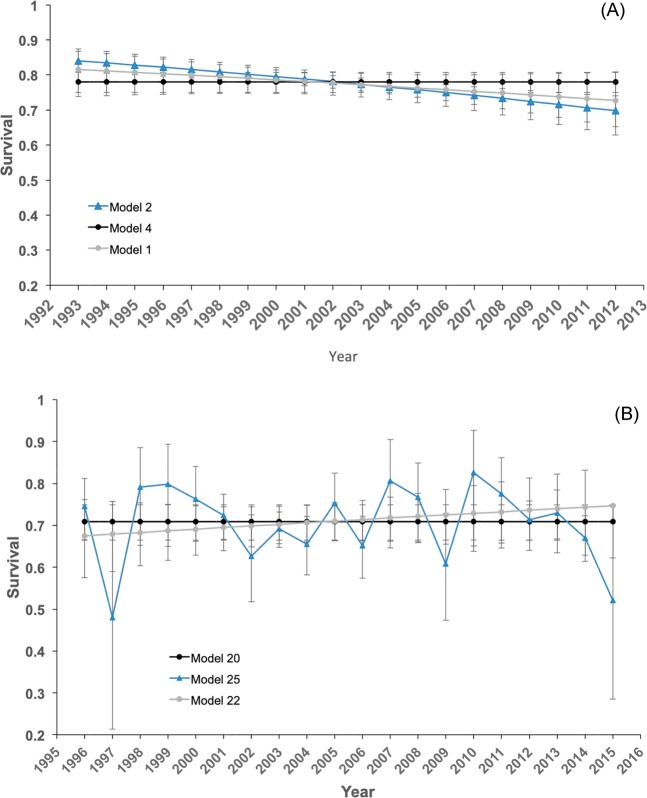
Changes in the annual survival probabilities of leatherback turtles (Mean and 95% CI) from the selected models in (**A**) Costa Rica (model 1 in grey, model 2 in blue and model 4 in black; Supplementary Table 1) and (**B**) México (Model 20, in black, model 22 in grey, and model 25 in blue; Supplementary Table 1). For Costa Rica survival show a decreasing trend over time in two of the best models and is constant in the other one. For México best models cannot disentangle whether survival is affected by Winter MEI, is constant, or show an increasing trend over time.

Probability of capture was lower and more variable in México (0.698, CI: 0.438–0.873) than in Costa Rica (0.887, CI: 0.772–0.948) (Table 1; Supplementary Table 1). We found differences in demographic parameters between the subpopulations of México and Costa Rica (Table 1; Supplementary Table 1). Adult survival was critically low in both subpopulations (annual survival probabilities for México = 0.705 [CI: 0.675–0.741] and Costa Rica = 0.778 [CI: 0.750–0.807]). To ensure population stability in long-lived species such as turtles, adult survival is generally 0.90 or higher^56,57^ with low variability over time^58^. Indeed, previous estimates of adult survival rates of nesting leatherback females have been ≥0.89 for nesting populations that were stable or increasing at the time of study^38–40^. The variation in adult survivorship estimates for México and Costa Rica could be related to higher rates of permanent dispersal by nesting leatherbacks from the index beaches in México than from the Costa Rican index beaches, or by differential mortality between subpopulations due to differential risk of bycatch (i.e., different fisheries affecting each nesting population), thus highlighting the need for targeted reduction efforts. Additionally, survival probabilities in Costa Rica showed substantial variation and an overall decrease over time, whereas in México annual survival did not vary much and increased slightly (Fig. 3). For México, one of the best models suggested a climatic effect on survival (Supplementary Table 1). A longer time series will allow us to better characterize climatic effects on survival in this species on both subpopulations.

Transient probabilities were high in both subpopulations (México = 0.279 [CI: 0.106–0.560]; Costa Rica = 0.239 [CI: 0.157–0.346]) suggesting that either there is a high permanent dispersal after the first breeding to other nesting beaches, or that in this species and population, there is a high cost of reproduction resulting in many individuals either dying or remaining at sea after their first breeding event. Climate might influence transient probabilities more strongly for México than for Costa Rica (Supplementary Table 1).

Remigration probabilities were similar between subpopulations but somewhat lower for México than Costa Rica (Table 1). Climatic factors seem to play a more important role driving remigration probabilities in México than in Costa Rica, at least in relation to the MEI (Supplementary Table 1).

### Bycatch estimates

Based on bycatch data compiled by members of the Laúd OPO Network, a minimum of 440 leatherback turtles (mostly adults, and some likely subadults; refs. ^27,31^) were caught by fisheries in the EP since 2012 (~97 turtles per year), of which 132 turtles were caught in longlines, 176 in gillnets, 100 in driftnets and 32 in other fishing gear or in non-specified gear.

The most robust on-board observer program in the region is in Chile’s industrial longline fleet, which has reported an average of 26 leatherback bycatch interactions per year (range 6 to 106) since 2001 (P. Zárate, Instituto del Fomento Pesquero Chile); however, mortality is very low in this fishery^31^. Additional data collected via rapid bycatch assessments of gillnet gear in Ecuador, Perú, and Chile^30^ provides estimates of up to 4,000 interactions with leatherbacks per year. However, these estimates should be viewed with caution because they are based on extrapolations from surveys to fleet-wide estimates of total fishing effort and reported interaction rates. Bycatch data we analyzed largely reflect small-scale fisheries bycatch because bycatch data for high-seas fisheries are generally unavailable.

### Population model

The estimated deterministic λ under current conditions was 0.863, reflecting a decline of about 15% per year in population size. When accounting for uncertainty, covariance between parameters, and environmental stochasticity in survival rates and under current conditions, the mean growth rate for the population λ_*s*_ under current conditions was 0.864 (95% CI: 0.834–0.896) (Table 2).

**Table 2:** Scenarios considered modifying adult/subadult and juvenile survivals and breeding propensities to simulate bycatch reduction.

Scenarios	1	2	3	4	5	6	7	8	9	10
Conservation efforts	Current (Status quo)	if there was no relocation	Egg harvest eradicated & increased emergence	Egg harvest eradicated & head-start	Bycatch reduced 5%, current beach protection	Bycatch reduced 10%, current beach protection	Bycatch reduced 20%, current beach protection	Bycatch reduced 5%, increased emergence	Bycatch reduced 10%, increased emergence	Bycatch reduced 20%, increased emergence
**Relocation**	yes	no	yes	yes	yes	yes	yes	yes	yes	yes
**Egg harvest eradicated**	no	no	yes	yes	no	no	no	no	no	no
**Increased emergence success**	no	no	yes	no	no	no	no	yes	yes	yes
**Headstarting**	no	no	no	yes	no	no	no	no	no	no
**Bycatch reduction**	no	no	no	no	5%	10%	20%	5%	10%	20%
**POPULATION RESPONSES**										
λ_*s*_	0.864	0.860	0.896	0.873	0.898	0.936	1.013	0.943	0.963	1.041
λ_*s*_ lower 95% CI	0.834	0.828	0.867	0.842	0.869	0.904	0.981	0.893	0.931	1.008
λ_*s*_ upper 95% CI	0.896	0.895	0.930	0.906	0.928	0.968	1.042	0.960	0.994	1.071
Time to extinction	59	57	79	64	80	99	—	96	—	—
Probability extinction in 100 yr	1	1	0.904	0.991	0.887	0.147	0	0.352	0.003	0

Responses were measured as stochastic population growth rate (λ_*s*_), time to extinction and probability of extinction in 100 years.

### Conservation scenarios

**Figure 4:**
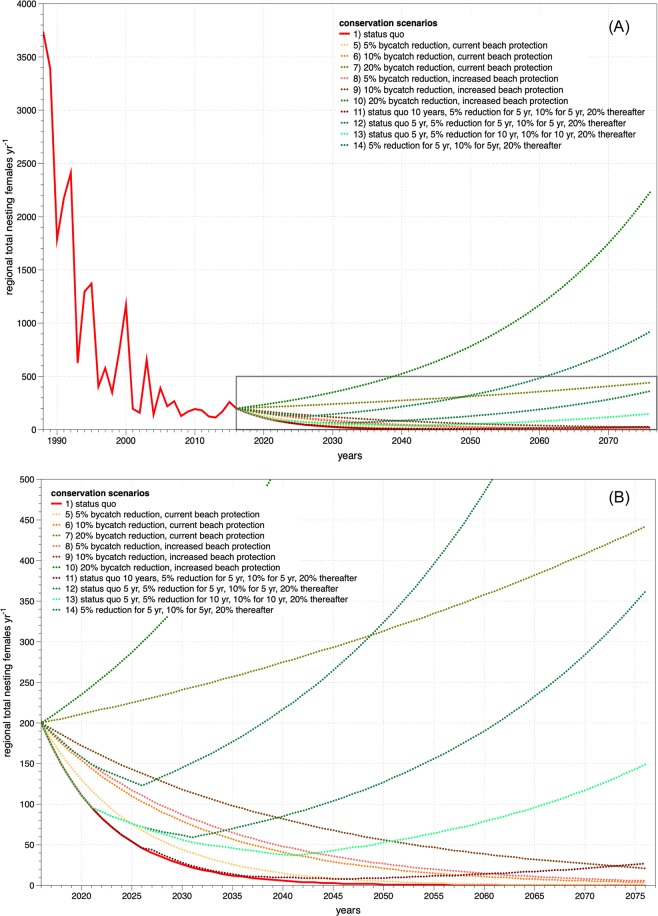
Modeled population projections under current and hypothetical conservation scenarios. Panel (A) shows data and projections from 1988 through 2076 (approximately three generations), while Panel (B) shows the area within the grey box (2016 through 2076; i.e., the next two generations) in more detail. Lines in red, orange, and yellow represent projections with negative population growth rates, while projections in green represent positive growth rate scenarios. The solid red line represents actual data through 2016, and future projections under status quo conditions.

Here we present results from 10 scenarios that best represent the various combinations of conservation effects on population growth rates (Fig. 4). The remaining scenarios not presented here offered slight permutations on the inputs described above and in Table 2 (e.g., intermediate values for bycatch reduction, differential changes in adult survivorship for Costa Rica/Nicaragua and México, fewer head-started individuals).

Time to extinction under current conditions (Scenario 1) is estimated at 59 years (Table 2; Fig. 4). Enhanced beach protection and improved nest conditions that favor increased hatchling production resulted in λ_s_ of nearly 0.90 (Scenario 3). In these scenarios, hatching success would be increased from 40% to ~60%, producing more than 7,000 additional female hatchlings per year, on average (see Eqs. 1 and 2).

Head-starting 50 individuals through first year of life (Scenario 4) did not increase λ_s_ over status quo (0.87 vs. 0.86) and had a negligible effect on time to extinction (64 years vs. 59 years). Effects of head-starting were less than if conservation efforts were to focus on eradication of human consumption of eggs and increased hatchling production by improving emergence success (λ_s_ = 0.90; Scenario 3) or on reducing adult mortality by 5% (λ_s_ = 0.90; Scenario 5) (Table 2; Fig. 4).

The only scenarios that predicted positive long-term population growth rates included 20% increase in late-stage survivorship (e.g., Scenarios 7 and 10; Table 2; Fig. 4). Scenarios that achieved full nest protection and increased emergence success as well as increased late-stage survivorship by 5% (Scenario 8) and 10% (Scenario 9) produced increased λ_s_ (0.94 and 0.96, respectively) and negligible probabilities of extinction in the next 100 years. However, these scenarios did not achieve stable or increasing population growth (Fig. 4).

Assuming that increased late-stage survivorship will not be possible immediately, we developed additional hypothetical scenarios that simulated stepwise increases in survivorship derived from other scenarios (e.g., λ_s_ associated with 5%, 10%, and 20% increases in survivorship) to examine the potential outcomes of delayed conservation gains that increase over time (Scenarios 11–14). Results of these projections demonstrate that survivorship must begin increasing in the next 5 years, and continue to increase thereafter until reaching at least 20%, for the population to avoid extinction. For example, Scenario 12 has 5 years of status quo survivorship, followed by 5 years at a 5% increase, years 6–10 at a 10% increase, and 20% increase thereafter (Fig. 4). If positive conservation outcomes are delayed by more than 10 years, the population will be unlikely to recover (Scenario 11, Fig. 4).

### Conservation implications

Our results show that increasing subadult and adult survival through fishery bycatch reduction is paramount to avoid the extinction of EP leatherbacks (e.g., Scenarios 7, 10, 12–14). Thus, redoubled, targeted conservation actions focused on reduction of bycatch mortality are urgently needed to reverse current population trends (Fig. 4).

To provide numerical targets for conservation efforts to reduce bycatch by 20%, we used two separate approaches. We acknowledge that the number of individuals saved with a 20% reduction in bycatch is not equivalent to a 20% increase in late-stage survivorship that was the actual model parameter. Nonetheless, there is conceptual validity to trying to understand the magnitude of the required reduction in bycatch if 20% of adults and subadults could survive another year.

The first approach relied on estimated numbers of leatherbacks taken as bycatch from the Laúd OPO Network and published data (e.g., ref. ^30^). Alfaro-Shigueto *et al*.^30^ estimated that nearly 47,000 sea turtles (all species) are taken annually as bycatch in net gears in Ecuador, Perú, and Chile, and that more than 16,000 of these incidents are lethal. Using port-specific estimates of leatherback interactions as a proportion of overall sea turtle interactions provided by Alfaro-Shigueto *et al*.^30^, we estimated that as many as 1,300 of these lethal interactions are leatherbacks, which include adult females and males, as well as subadults, and also includes leatherbacks from the West Pacific leatherback subpopulation (~15% of the total number; ref. ^59^). We also estimated leatherback bycatch and mortality in terms of mortality per boat per year for each port surveyed, and arrived at similar estimates of total mortality (~1,100 individuals). Based on these estimates, a 20% reduction in bycatch mortality would mean preventing mortality of between 200 and 222 leatherbacks per year.

The second approach used stage-based abundance estimates from the model-derived stable age distribution that emerged from our population model to estimate annual late-stage mortality currently and under a 20% increase in survivorship. Based on the nesting numbers through 2015, there are approximately 1,678 subadults and adults (1,398 females, assuming that operational sex ratio is the same as estimated sex ratio) in the entire population. Applying the current survivorship estimate for Costa Rica (0.78), approximately 369 adult and subadult turtles (308 of which would be females) die annually. Then, we calculated annual mortality using the survivorship estimate under which the population will increase (0.94), which is approximately 107 individuals (89 females). The difference, 262 leatherbacks (218 females), is the number that would survive under the recovering population scenario that would have died under current conditions. In other words, a 20% increase in late-stage survivorship would mean saving an additional 262 leatherbacks per year above the current scenario.

Both approaches relied on significant assumptions that are worth noting. First, in the case of mortality estimates from Alfaro-Shigueto *et al*.^30^ and the Laúd OPO Network, we used extrapolations to fleet-wide estimates based on reported bycatch reflecting from a subset of fishing effort, a practice that, while conceptually defensible, often is unable to incorporate the true relationship between the sample data and the entire fleet, nor all of the factors that drive these relationships^26^. For example, comparing the estimates of total mortality (>1,000) to the total number of subadults and adults in the population based on the stable age distribution (<1,700) indicates that the former are apparently unrealistic. Nonetheless, this approach can provide context and scale for the actual amount of bycatch occurring and by how much it needs to be reduced.

As for the second approach, assuming that a severely depleted population that is still declining has a stable age or stage structure is obviously tenuous. The numbers generated by this exercise are dependent on using annual averages of nesting females. In the years subsequent to the period used for the mark-recapture histories, higher numbers of nesting females were observed in México (Fig. 1). If these more recent, higher values were used in the population model instead of the 176 total females estimated between 2010 and 2015, our estimated number of adult and subadult leatherbacks would also be higher. The same dynamic affects the calculation of survivorship if a higher number of remigrant turtles returned to nest in the most recent years than did during the study period. Moreover, if we assume that the mortality estimated from bycatch assessments described above^30^ are closer to the true values than the estimated number of late-stage leatherbacks currently in the population, this would imply that there are more leatherbacks across life stages than the stable age distribution from the model estimated. Prolonged juvenile stages and delayed reproduction among subadults and adult-aged leatherbacks might mean that there are far more individuals in these life stages than we have estimated. In addition, operational sex ratios in adult turtles might be less female-skewed than primary sex ratios of hatchlings, as demonstrated for Caribbean leatherbacks^60^.

While keeping these issues in mind, we conducted this exercise to provide targets to guide individual conservation efforts toward a shared, regional conservation goal. Two independent approaches yielded similar estimates of the numbers of late-stage leatherbacks that need to be saved to meet regional population targets (i.e., 20% increase in survivorship, or approximately 200 to 262 adult and subadult leatherbacks). Regardless of what the exact number of turtles to be saved is, what is clear is that late-stage leatherback survivorship must be significantly increased over current rates for the population to stabilize and eventually recover (Fig. 4).

It is worth noting that the above exercise does not include other anthropogenic sources of mortality (i.e., ingestion of plastic debris, deliberate take of adult turtles). Further, bycatch estimates, as well as estimates of current and future bycatch under improved conservation outcomes, are biased toward availability of data from small-scale fisheries. However, increased late-stage survivorship must reflect bycatch reduction across all fisheries in which leatherback mortality occurs (and other mortality sources), including industrial-scale longlines, not just small-scale operations within countries.

While efforts to increase late-stage survivorship will have the most significant impact on recovering the EP leatherback population, enhanced conservation efforts on nesting beaches can accelerate recovery. For example, increasing production of hatchlings by eliminating egg consumption by humans and improving incubation conditions for embryonic development could produce 7,000 to 8,000 more hatchlings per year, based on reproductive parameters shown in Table 1. Combining this enhanced hatchling production with increased adult survivorship by 20% (Scenario 10) could result in a 3% faster population growth rate than if adult survivorship alone were increased and hatchling production remained unchanged (Scenario 10 λ_s_ = 1.04; vs Scenario 7 λ_s_ = 1.01; Table 2). Thus, sustaining nesting beach conservation efforts at current levels is fundamentally important, and increasing those efforts to produce more hatchlings will enhance population recovery.

### Conclusions and next steps

Here we have reported the first population analysis and projections for the entire EP leatherback metapopulation. Results of our analysis show that the prognosis for this population is dire: under current conditions, the population remains on a path toward regional extinction (Fig. 4) in the next 60 years, and likely sooner in Nicaragua and Costa Rica (Fig. 1). However, our model shows that if conservation efforts that are targeted and scaled appropriately toward high-priority sites, and if projects are implemented as soon as possible and maintained over time, the EP leatherback population trend can eventually stabilize and increase. The case study of the Kemp’s ridley sea turtle (*Lepidochelys kempii*) provides a relevant comparison to the EP leatherback situation. Kemp’s ridleys declined precipitously into the 1970s before a concerted, coordinated, bi-national effort between government authorities in México and the USA established and implemented actions to address the highest impact threats: harvest of adult females and eggs and fisheries bycatch mortality^61^. Although the current Kemp’s ridley population size is still a small fraction of its historical abundance^62^, these efforts saved the species from extinction and promoted a long-term population increase.

Overall, our results underscore the urgency of increased conservation efforts both in marine environments to improve late-stage survivorship through significant reduction of bycatch mortality, as well as on nesting beaches to maintain and enhance (where possible) activities that produce more hatchlings. Such efforts could include:

- expanded capacity-building in coastal fisheries for safe handling and release of bycaught leatherbacks;
- testing and implementation of bycatch mitigation measures in all fisheries that have interactions with leatherbacks (i.e., modifications to fishing gear that reduce bycatch mortality);
- incentivization of small-scale fishers to reduce bycatch, including market-based opportunities that deliver increased value for products fished by methods that minimize environmental damage such as bycatch;
- implementation of actions to monitor and reduce leatherback bycatch in fisheries that interact with leatherbacks, particularly those operating in high-seas areas within the Convention Area of the Inter-American Tropical Tuna Commission (IATTC), including (but not limited to) enhanced observer coverage and implementation of best practices for safe handling and release of sea turtles, potentially targeted in leatherback high-use areas identified by satellite telemetry^23,24^ and other information sources^27–31,49^ (Fig. 1);
- enhanced protection of leatherback nests from human consumption and beach erosion;
- improved nest relocation practices to promote incubation conditions within leatherback nests that favor increased production of hatchlings and promote population growth, while accounting for potential climate change effects^18,19^;
- strategic coordination between the Laúd OPO Network and national authorities and intergovernmental bodies (e.g., IATTC, Inter-American Convention for the Protection and Conservation of Sea Turtles, Comisión Permanente del Pacífico del Sur, Convention on Migratory Species) to ensure alignment of priority actions for leatherback recovery are reflected and implemented through existing governance mechanisms.

Fortunately, many of these activities are currently ongoing in various places in the region^35^. In fact, the IATTC recently approved strengthened measures to monitor and reduce effects of bycatch on leatherbacks and other sea turtle species in the Eastern Pacific region^63^. To meet the challenge of saving EP leatherbacks from extinction, stakeholders from governments, non-governmental organizations, local communities, and research institutions must collaboratively expand, sustain, and coordinate high-priority, impactful conservation efforts at local, national, and international scales throughout the Eastern Pacific Ocean region.

## Supplementary information

Supplementary Information.
