# Supplementary material for: Enhanced, coordinated conservation efforts required to avoid extinction of critically endangered Eastern Pacific leatherback turtles
Source: Sci Rep. 2020 Mar 16;10:4772. doi: 10.1038/s41598-020-60581-7 (PMC7075994; doi:10.1038/s41598-020-60581-7)
Supplement: Supplementary file 1 — Supplementary Information. [file 41598_2020_60581_MOESM1_ESM.docx]

**Supplementary Table 1.** Model selection for assessing factors affecting demographic parameters of the two subpopulations. The dataset extends between 1996 and 2016 for México and between 1993 and 2013 for Costa Rica. The best models for each subpopulation are marked in bold. Models used to get the variance-covariance matrix between transient probability, survival probability and breeding propensity probabilities are shown in italics. Tr is the probability of being a transient, Phi, the survival probability, B, the probability of remigrating after 1,2,3 and 4 or more years, p is the probability of capture; Parameters were kept constant (.) or were allowed to change with time (t); ‘T’: a trend over the study period; ‘w’: effect of the Winter MEI; * represents and interaction between terms; + represents an additive term

| **Model** | **Tr** | **Phi** | **B** | **p** | **np** | **Dev** | **QAICc** | **Δ AIC** | **AIC W** |
| --- | --- | --- | --- | --- | --- | --- | --- | --- | --- |
| **México** |  |  |  |  |  |  |  |  |  |
| 1 | T | T | 4+t | . | 28 | 2697,47 | 1703,48 | 2,410 | 0,094 |
| 2 | . | T | 4+t | . | 27 | 2702,02 | 1704,21 | 3,138 | 0,091 |
| 3 | . | . | 4+t | . | 26 | 2706,86 | 1705,12 | 4,049 | 0,058 |
| 4 | T | . | 4+t | . | 27 | 2706,55 | 1706,98 | 5,908 | 0,023 |
| 5 | . | . | 4 | t | 26 | 2716,69 | 1711,12 | 10,051 | 0,003 |
| 6 | . | T | 4 | . | 8 | 2794,03 | 1721,82 | 20,749 | 0,000 |
| 7 | T | . | 4 | . | 8 | 2795,66 | 1722,81 | 21,743 | 0,000 |
| 8 | . | . | 4 | . | 7 | 2804,12 | 1725,96 | 24,891 | 0,000 |
| 9 | . | t | 4 | . | 26 | 2743,37 | 1727,41 | 26,340 | 0,000 |
| 10 | t | . | 4+t | . | 45 | 2683,82 | 1730,19 | 29,126 | 0,000 |
| 19 | . | t | 4+t | . | 45 | 2688,99 | 1733,35 | 32,283 | 0,000 |
| 11 | T | T | 4+t | t | 47 | 2683,92 | 1734,41 | 33,341 | 0,000 |
| 12 | . | T | 4+t | t | 46 | 2688,90 | 1735,38 | 34,307 | 0,000 |
| 13 | . | . | 4+t | t | 45 | 2692,58 | 1735,55 | 34,477 | 0,000 |
| 14 | t | . | 4 | . | 26 | 2785,43 | 1753,09 | 52,019 | 0,000 |
| 15 | . | t | 4+t | t | 62 | 2675,30 | 1760,54 | 59,474 | 0,000 |
| 16 | . | T | 4*t | . | 76 | 2665,06 | 1783,95 | 82,884 | 0,000 |
| 17 | . | . | 4*t | . | 75 | 2674,72 | 1787,72 | 86,650 | 0,000 |
| 18 | . | t | 4*t | t | 91 | 2651,28 | 1807,71 | 106,642 | 0,000 |
| 19 | . | t | 4+t | . | 45 | 2688,99 | 1733,35 | 32,283 | 0,000 |
| **20** | **w** | **.** | **4*w** | **t** | **31** | **2683,45** | **1701,07** | **0,000** | **0,438** |
| 21 | . | w | 4*w | t | 31 | 2693,94 | 1707,47 | 6,401 | 0,018 |
| **22** | **w** | **T** | **4*w** | **t** | **32** | **2682,17** | **1702,34** | **1,271** | **0,232** |
| 23 | w | . | 4*w | . | 12 | 2762,38 | 1710,56 | 9,496 | 0,004 |
| *24* | *.* | *.* | *4* | *t* | 26 | 2716,69 | 1711,12 | 10,051 | 0,003 |
| **25** | **w** | **w** | **4*w** | **t** | **32** | **2680,432** | **1701,279** | **0,210** | **0,394** |
| **Costa Rica** |  |  |  |  |  |  |  |  |  |
| **1** | **T** | **T** | **4+t** | **.** | **28** | **6094,99** | **2052,97** | **0,958** | **0,265** |
| **2** | **.** | **T** | **4+t** | **.** | **27** | **6098,29** | **2052,01** | **0,000** | **0,428** |
| 3 | . | . | 4+t | . | 26 | 6118,93 | 2056,74 | 4,723 | 0,040 |
| **4** | **T** | **.** | **4+t** | **.** | **27** | **6101,19** | **2052,96** | **0,949** | **0,266** |
| *5* | *.* | *.* | *4* | *t* | *27* | *6208,92* | *2088,25* | *36,236* | *0,000* |
| 6 | . | T | 4 | . | 8 | 6444,10 | 2126,79 | 74,779 | 0,000 |
| 7 | T | . | 4 | . | 8 | 6454,07 | 2130,06 | 78,047 | 0,000 |
| 8 | . | . | 4 | . | 7 | 6455,21 | 2128,42 | 76,406 | 0,000 |
| 9 | . | t | 4 | . | 26 | 6307,02 | 2118,35 | 66,332 | 0,000 |
| 10 | t | . | 4+t | . | 45 | 6068,04 | 2079,06 | 27,041 | 0,000 |
| 11 | T | T | 4+t | t | 47 | 6067,04 | 2082,86 | 30,848 | 0,000 |
| 12 | . | T | 4+t | t | 46 | 6070,24 | 2081,84 | 29,829 | 0,000 |
| 13 | . | . | 4+t | t | 45 | 6084,04 | 2084,30 | 32,281 | 0,000 |
| 14 | t | . | 4 | . | 26 | 6416,77 | 2154,29 | 102,277 | 0,000 |
| 15 | . | t | 4+t | . | 63 | 6041,03 | 2107,64 | 55,622 | 0,000 |
| 16 | . | T | 4*t | . | 78 | 6112,13 | 2162,49 | 110,476 | 0,000 |
| 17 | . | . | 4*t | . | 77 | 6015,16 | 2128,61 | 76,600 | 0,000 |
| 18 | . | t | 4*t | t | 108 | 5969,37 | 2179,91 | 127,896 | 0,000 |
| 19 | . | t | 4+t | . | 45 | 6059,77 | 2076,35 | 24,333 | 0,000 |
| 20 | w | . | 4*w | t | 31 | 6186,27 | 2089,00 | 36,988 | 0,000 |
| 21 | . | w | 4*w | t | 31 | 6183,81 | 2088,19 | 36,180 | 0,000 |
| 22 | . | T | 4*w | t | 31 | 6167,25 | 2082,77 | 30,757 | 0,000 |
| 23 | T | T | 4*w | t | 32 | 6165,70 | 2084,31 | 32,296 | 0,000 |
| 24 | T | T | 4*w | . | 13 | 6415,24 | 2127,42 | 75,405 | 0,000 |
